# Cancer cells exhibit clonal diversity in phenotypic plasticity

**DOI:** 10.1098/rsob.160283

**Published:** 2017-02-15

**Authors:** Robert Austin Mathis, Ethan S. Sokol, Piyush B. Gupta

**Affiliations:** 1 Whitehead Institute for Biomedical Research, 455 Main Street, Cambridge, MA 02142, USA; 2 Department of Biology, Massachusetts Institute of Technology, Cambridge, MA 02139, USA; 3 Koch Institute for Integrative Cancer Research at MIT, Cambridge, MA 02139, USA; 4 Harvard Stem Cell Institute, Cambridge, MA 02138, USA

**Keywords:** phenotypic plasticity, tumour heterogeneity, cell state, clonal evolution

## Abstract

Phenotypic heterogeneity in cancers is associated with invasive progression and drug resistance. This heterogeneity arises in part from the ability of cancer cells to switch between phenotypic states, but the dynamics of this cellular plasticity remain poorly understood. Here we apply DNA barcodes to quantify and track phenotypic plasticity across hundreds of clones in a population of cancer cells exhibiting epithelial or mesenchymal differentiation phenotypes. We find that the epithelial-to-mesenchymal cell ratio is highly variable across the different clones in cancer cell populations, but remains stable for many generations within the progeny of any single clone—with a heritability of 0.89. To estimate the effects of combination therapies on phenotypically heterogeneous tumours, we generated quantitative simulations incorporating empirical data from our barcoding experiments. These analyses indicated that combination therapies which alternate between epithelial- and mesenchymal-specific treatments eventually select for clones with increased phenotypic plasticity. However, this selection could be minimized by increasing the frequency of alternation between treatments, identifying designs that may minimize selection for increased phenotypic plasticity. These findings establish new insights into phenotypic plasticity in cancer, and suggest design principles for optimizing the effectiveness of combination therapies for phenotypically heterogeneous tumours.

## 1. Background

The diversity of cancer cell phenotypes within individual tumours plays a major role in driving both drug resistance and tumour progression [1,2]. For decades, the prevailing view has been that phenotypic diversity arises because tumours are mixtures of cancer cell clones with distinct yet heritable phenotypes. In this neo-Darwinian model, cancer cell phenotypes are genetically encoded and thus stably propagated to daughter cells [3–6]. In support of this model, there are significant genetic differences between different sections of a tumour, and even across different cells from the same tumours [5–9].

Phenotypic heterogeneity has been documented in breast tumours and breast cancer cell lines [10,11]. Several recent reports have suggested that there are bi-directional transitions between cancer cells in distinct phenotypic states for various kinds of cancers [12–22]. For example, breast cancer cells in culture transition between mesenchymal (stem-like) and epithelial (differentiated) states [12–14,16,17,23]. Analyses of cells within patient tumours also suggest that they transition between phenotypic states [18,24]. In any population, random transitions of cells between phenotypic states will give rise to a stable equilibrium in which the different phenotypic states are represented at fixed proportions [12].

Since phenotypic plasticity has primarily been examined in populations of cancer cells, it is currently not known if this trait varies across the different cancer cell clones within a single population. Genetic analyses of phenotype states sorted from tumours and cell lines have led to conflicting conclusions regarding the contribution of genetic mutations to phenotypic plasticity [24–27]. While some studies have confirmed clonal relationships between states, a key question that remains open is if phenotypic plasticity can vary across the clones in a single cancer cell population [28].

Resolving this question—whether the clonal diversity of cancers influences their phenotypic plasticity—is fundamental to understanding cancer, and is also important from the perspective of developing combination cancer therapies. In particular, optimal combination chemotherapy designs will depend on whether the clones in a tumour have different capacities to transition between drug-sensitive and -resistant states.

Examining this question would require an experimental approach that can quantify phenotypic plasticity in hundreds of individual clones within a population of cancer cells. DNA barcodes combined with high-throughput sequencing have proven effective for tracking large numbers of clones in both normal and cancer cell populations [28–30]. Here, we apply DNA barcodes to quantify the extent to which phenotypic plasticity varies across hundreds of clones within a single population of cancer cells.

## 2. Results

### 2.1. Labelling of cancer cell clones with DNA barcodes

**Figure 1.**
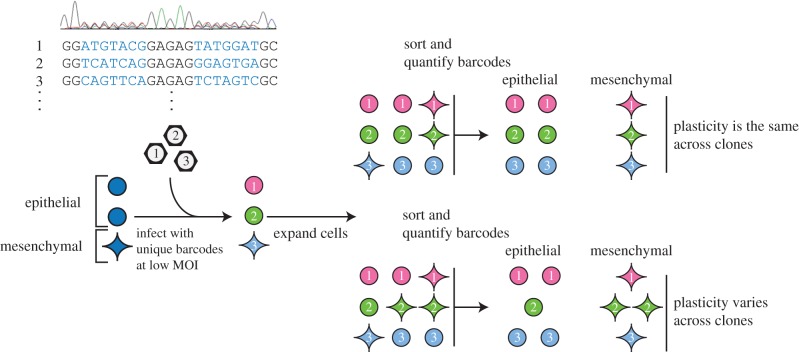
DNA barcode strategy to examine plasticities across clones. DNA barcodes, stably introduced into cells, can be used to track the progeny of individual cells. Sorting these cells, and quantifying the abundance of each barcode in both cell states, will distinguish if clones vary in phenotypic plasticity. Included in the schematic is a Sanger sequence trace of the pool of barcode plasmids.

To track the progeny of single cancer cells, we used retroviruses to stably introduce a random DNA sequence (or barcode) into their genome. These barcodes were introduced into 1 × 10^4^ MDA-MB-157 cells at a low multiplicity of infection (0.13), which we expected would label approximately 1300 individual clones (electronic supplementary material, figure S1*a*). After a brief drug selection for the infected cells, the barcoded clones were expanded in culture over a span of several months (figure 1). Since the retrovirus pool contains approximately 2.6 × 10^6^ random barcodes (electronic supplementary material, figure S1*b*), and only approximately 1300 cells were infected, there was a probability of 0.31 that more than one cell was independently infected with the same barcode, and a probability of 6.1 × 10^−3^ that four or more cells shared a barcode with other cells (electronic supplementary material, figure S1*c*). Accordingly, the number of copies of a given barcode sequence in the genomic DNA is directly proportional to the size of the corresponding clone in the population.

High-throughput sequencing of the barcodes from the pool of clones revealed that the barcodes were well-separated in DNA sequence space, with an average pair-wise Hamming distance of 10.5 base pairs. This is consistent with what one would expect if 1372 DNA sequences of length 14 were randomly sampled from a space of 300 million possible sequences. Since the barcodes were well-separated in sequence space, it was straightforward to map reads to barcodes even in cases where point mutations arose through sequencing, consistent with the findings of others [28,31,32]; such reads were an average of 1.7 base pairs from their parent barcodes.

### 2.2. Clones have heterogeneous phenotypic ratios

We chose to barcode the MDA-MB-157 cell line because this line contains both epithelial and mesenchymal phenotypic states that can be robustly separated by fluorescence-activated cell sorting (FACS). Using an antibody that recognizes keratins 8 and 18, intracellular antigens which mark luminal epithelial cells in the mammary gland [33], we were able to separate the cells into keratin 8/18 high or low fractions (electronic supplementary material, figure S2*a,b*). Importantly, this population of cells also contained roughly equal amounts of the two phenotypic states, with about 40% mesenchymal cells. Having a large minor population meant we were confident we could accurately detect clones with small amounts of progeny in the minor state.

**Figure 2.**
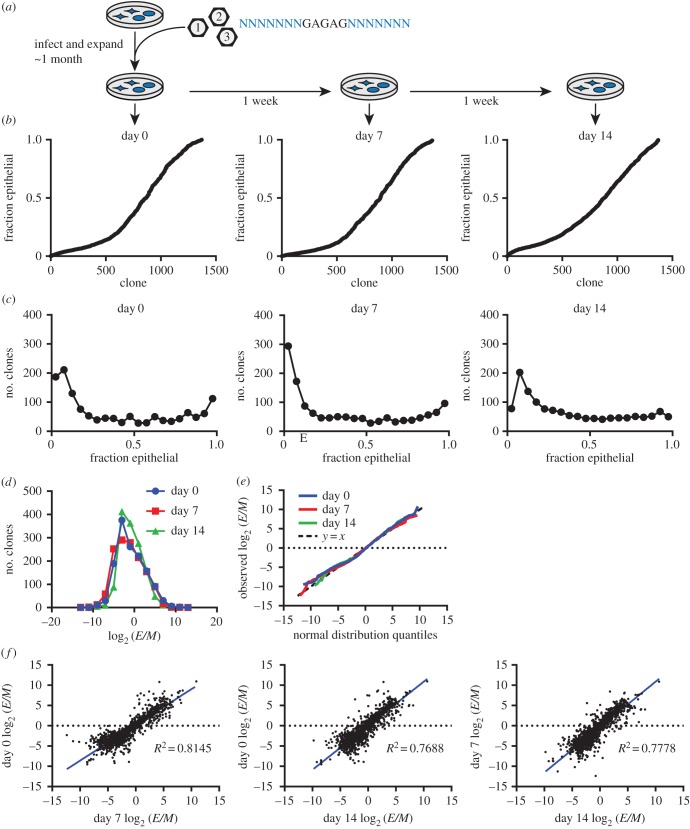
Phenotypic plasticity varies across clones. (*a*) Cells stably transduced with DNA barcodes were expanded over approximately 1 month into a large population. Three times over 2 weeks, samples of the population were sorted by cell state, and each clone's representation was quantified in these sorted states. (*b*) The fraction of each clone in the epithelial state (fraction epithelial) is plotted for each time point, showing the diversity of phenotypic plasticities among clones. Clones are sorted in ascending order by their fraction epithelial, calculated from the mean clone size in duplicate sorts. (*c*) Histograms of clones, binned by the fraction of each clone that is epithelial, show the distribution of clonal plasticity. Each plot is from a different time point. (*d*) Histogram of clones binned by their log_2_ ratio of epithelial (*E*) to mesenchymal (*M*) cells (log_2_ (*E*/*M*)), with one plot for each time point, showing phenotypic plasticity is approximately log normally distributed across clones. (*e*) Quantile/quantile plot with the distribution of log_2_ (*E*/*M*) across clones plotted against the quantiles of a normal distribution with the same mean and standard deviation, with one line for each time point. Also plotted is the line *y* = *x* (dashed line), representing a perfect normal distribution. (*f*) Each clone's log_2_ (*E*/*M*) in one time point plotted against its log_2_ (*E*/*M*) in another time point, showing that clones have stable phenotypic plasticity. The *R*^2^ (squared Pearson correlation coefficient) is shown, and a linear regression of the data is plotted in blue. Each clone's log_2_ (*E*/*M*) was calculated from the mean clone size in duplicate sorted *E* and *M* states.

To assess the proportion of cells with epithelial or mesenchymal phenotypes within each clone, we separated the barcoded population into epithelial and mesenchymal fractions with FACS (figure 1). To assess clonal dynamics, the same population of cells was sampled once weekly for a total of three time points, each time separating these phenotypes (figure 2*a*). After sorting, each population was further divided into equal halves before extracting and sequencing its DNA. Using high-throughput sequencing, we quantified the proportion of cells with epithelial and mesenchymal phenotypes for each of the 1372 barcoded clones in the population.

To estimate the magnitude of the technical error associated with sample preparation, sequencing and analysis, we compared estimated clone sizes between each of the two sequenced partitions of these six populations (two sorted populations at three time points). The clone size estimated for each barcode was highly reproducible between these technical replicates, with an average Pearson correlation of 0.9119 across the 1372 clones detected (electronic supplementary material, figure S1*d*). These observations indicated that this experimental approach reproducibly quantified the numbers of cells corresponding to barcoded clones within the population.

Ordering clones by their fraction of epithelial cells revealed that the majority of clones produced progeny that were mixtures of cancer cells in the two phenotypic states (figure 2*b*, electronic supplementary material, table S1), with only 11% of clones consisting of only one lineage (statistically indistinguishable from mis-sorted cells). Although most clones exhibited such phenotypic plasticity, the ratio of epithelial to mesenchymal phenotypes varied significantly between clones (Shannon entropy = 3.5).

From the sequencing data we were able to distinguish three distinct classes of clones: clones with mostly epithelial cells, clones with mostly mesenchymal cells, and clones that were a mixture of cells in these two phenotypic states (figure 2*c*). The majority of clones (89%) in the population gave rise to daughter cells in both the mesenchymal and epithelial states, with slightly more than half of the clones (64%) having a mesenchymal bias. We observed that the distribution of epithelial-to-mesenchymal ratios across clones was closely approximated by a log-normal distribution (figure 2*d,e*), for all three time points. While most clones comprised both epithelial and mesenchymal cells, the proportion of progeny in these two states varied greatly between clones: 93% of clones had a bias significantly different from the bulk population proportions of the two states.

Although most clones had a mesenchymal bias, epithelial-biased clones tended to be larger (electronic supplementary material, figure S3*a*), resulting in approximately 60% of the population of cells being epithelial. Despite this difference, there was only a weak correlation (0.06) between a clone's growth rate and log_2_ (*E*/*M*) ratio, although we found the differences in clones' growth rates to be stable across the time course (electronic supplementary material, figure S3*b–d*).

### 2.3. Phenotypic ratios are stably inherited by clonal progeny

**Figure 3.**
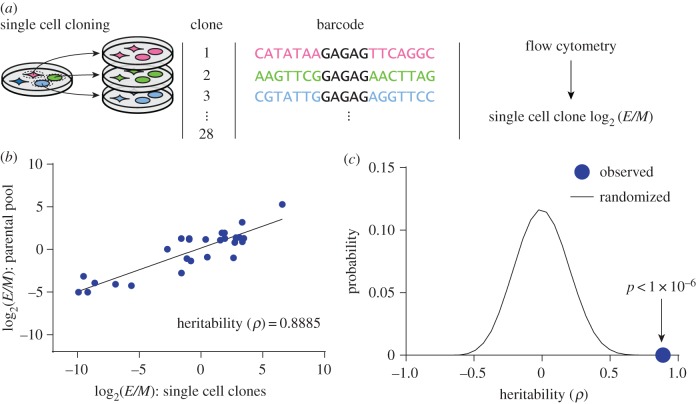
Phenotypic plasticity is stably inherited. (*a*) Clonal subpopulations were generated from single cells, and each clone's phenotypic ratio was evaluated with flow cytometry. (*b*) Each single-cell clone's log_2_ ratio of epithelial to mesenchymal cells (log_2_ (*E*/*M*)) is plotted against the log_2_ (*E*/*M*) of the same clone in the parental pooled population. The Pearson correlation coefficient is shown, estimating the narrow-sense heritability. In black is a linear regression of the data. Phenotypic ratio is a heritable phenotype. (*c*) Barcode labels were randomized 10^6^ times, and the Pearson correlation was calculated for each iteration; the observed correlation (blue circle) was higher than all of the randomizations, suggesting the heritability of phenotypic ratio is not likely due to random chance.

Although phenotypic ratios varied significantly across clones, they were highly stable for any given clone during the 2 weeks in culture, with an average Pearson correlation of 0.89 (*R*^2^ = 0.79, all *p* < 1 × 10^−6^) (figure 2*f*). Additionally, 81% of clones' fraction epithelial differed by less than 0.15 over 2 weeks. This raised the possibility that the epithelial-to-mesenchymal ratio could be a quantitatively inherited phenotype. To quantify the narrow-sense heritability of this trait, we generated 28 clonal subpopulations from individual cells expanded in culture over a span of 6 weeks. After expanding these clonal subpopulations, we used Sanger sequencing to determine their DNA barcodes. We also used flow cytometry to determine the phenotypic ratio in each of the cloned subpopulations, and compared this with the phenotypic ratio of the same clone in the parental pooled population (figure 3*a*, electronic supplementary material, figure S4 and table S2). Regression analysis of these comparisons indicated that phenotypic plasticity was a highly heritable trait (*ρ* = 0.89) (figure 3*b*). This heritability was considerably higher than that observed with 10^6^ datasets with permutated barcode labels that randomized the relationship between the parental and cloned populations (figure 3*c*). This finding indicated that phenotypic plasticities were stably inherited even through the rigors of single-cell cloning.

### 2.4. Phenotypic plasticity varies across clones in primary tumours

**Figure 4.**
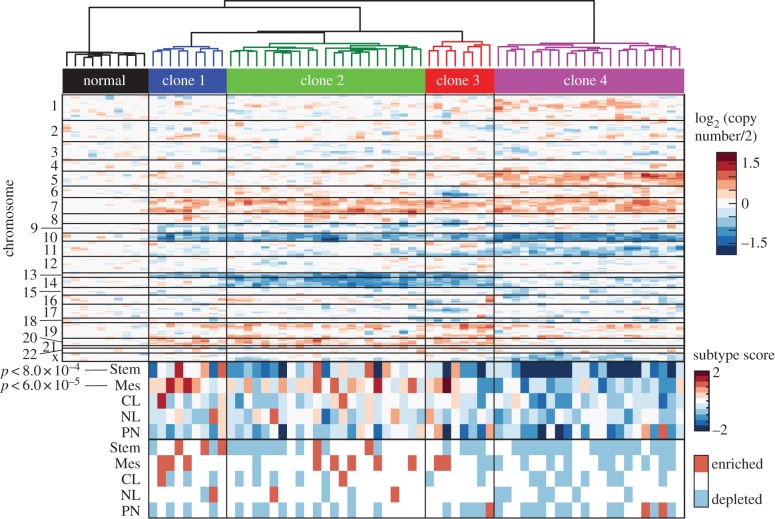
Phenotypic plasticity varies across clones *in vivo*. At the top is a heat map of copy-number variation of single cells estimated from single-cell RNA sequencing data of a primary glioblastoma [18]. Shown is a heatmap of the averaged, normalized expression of a sliding window of 100 genes moving across chromosomes, revealing chromosomal gains and losses. Each value shows the estimated log_2_ (copy number/2) for genes in the window. Based on these data, clones were grouped by hierarchical clustering based on Ward clustering of the Euclidean distances between clones, shown above. Below, each cell was given a score based on the average expression of a set of classifier genes for different tumour subtypes [35] and a score for glioblastoma stemness [18], shown as a heatmap. CL, classical; NL, neural; PN, proneural; Stem, stem-like; Mes, mesenchymal. Kruskal–Wallis tests showed differential representation of the mesenchymal (Mes) subtype (*p* < 6 × 10^−5^) and the stemness score (Stem) (*p* < 8 × 10^−4^) among clones. At the bottom, each cell's subtype scores are evaluated for significance compared with the background of gene expression in that cell. Scores higher or lower than 95% of gene sets were marked as enriched or depleted.

To assess whether phenotypic plasticity varies across clones in patient tumours, we analysed data from a recently published study that performed RNA sequencing on single primary tumour cells [18]. By identifying clonal relationships between cells, and determining cell states, we could test whether these clones also had different cell-state proportions. To identify clonal relationships, we looked for chromosomal gains and losses using a sliding average of gene expression moving across chromosomes, modified from published methods [18,34]. Although this limited resolution of genetic aberrations means we are likely to be missing genetic differences between clustered cells, we are confident the gains and losses of whole chromosomes reveal distinct clones. This analysis revealed a common gain in chromosome 7 and loss of chromosome 10 across tumour cells, aberrations commonly found in glioblastoma [35], as well as other changes, such as a gain of chromosome 5 or a loss of chromosome 14 or 13, that were only present in some cells (figure 4). Hierarchical clustering grouped single cells into clones based on these inferred chromosomal gains and losses, resulting in four major clones (figure 4).

We used the same single-cell RNA sequencing data to assign cells to cell states, using a published method based on the mean expression of gene sets defining different glioblastoma subtypes [18,35], and increased ‘stemness’ [18] (figure 4). This analysis revealed that while each clone contained cells representing different glioblastoma subtypes, there were significant differences in subtype scores between clones, particularly of the mesenchymal subtype (*p* < 6 × 10^−5^) and a stem-like state (*p* < 8.0 × 10^−4^). Although this snapshot in time cannot tell us about the stability of these differences, this result suggests that clones within primary tumours have different cell-state proportions, consistent with our previous observations.

### 2.5. Combination chemotherapies enrich for clones with increased phenotypic plasticity

While there is significant interest in developing combination therapies that incorporate agents which selectively target the epithelial and mesenchymal states, the optimal design of such therapies is likely to depend on the mechanisms that give rise to phenotypic diversity in tumours. We therefore used computational simulations to model how such chemotherapies would affect tumours that are heterogeneous mixtures of clones with different phenotypic plasticities. These tumours were simulated to match the plasticities, sizes and growth rates of the observed clones.

**Figure 5.**
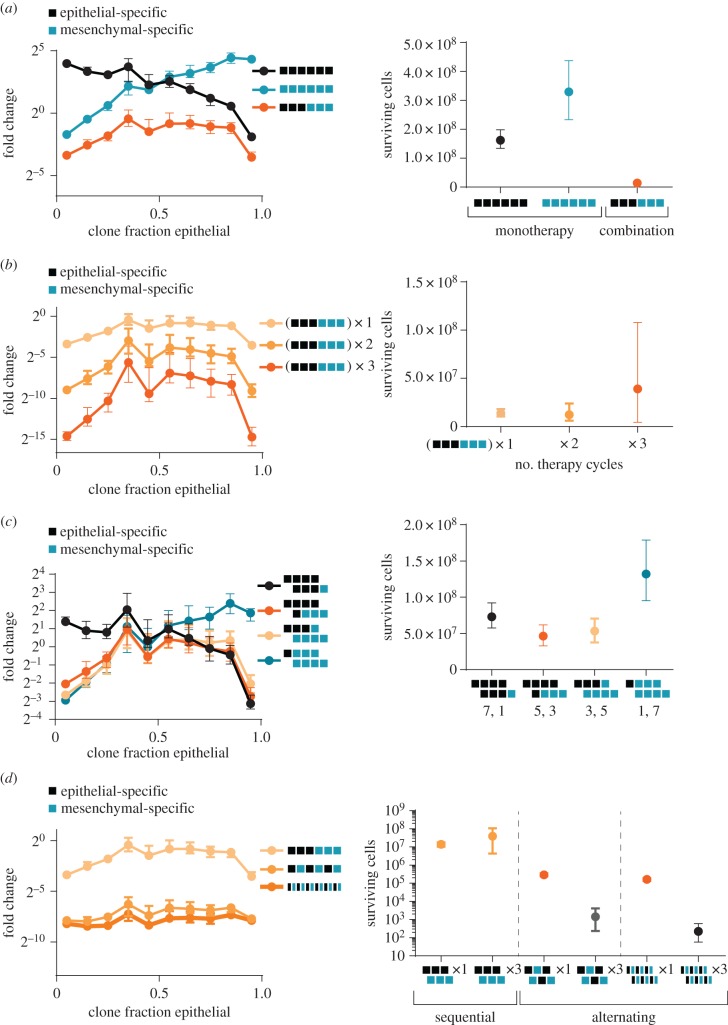
Combination chemotherapy enriches for clones with increased phenotypic plasticity. Simulations of clones treated with different patterns of combination therapies that include mesenchymal-specific (blue squares) and epithelial-specific treatments (black squares) (see Material and methods). (*a–d*) Left, the median fold change in clone size during the course of treatment for clones binned by the fraction of their progeny in the epithelial state. Displayed is the median and 90%–10% range of observed medians across 500 simulations. Right, the number of cancer cells surviving at the end of the simulation for each treatment; displayed is the median and 90%–10% range of observed cell numbers across 500 simulations. (*a*) Combination therapy (orange curve) enriches for clones with increased plasticity, while monotherapies enrich for clones in predominantly one or the other state. Fewer cells survive combination therapy. (*b*) Increasing the cycles of combination therapies (as shown in (*a*)) further enriches for clones with increased plasticity. However, resistant populations eventually emerge. The number of cycles of each combination therapy is indicated. (*c*) Different patterns of combination therapy, with varying proportions of epithelial- and mesenchymal-specific treatments, enrich for different, particular plasticities. (*d*) More rapid alternation between therapies reduces the enrichment for more plastic clones and more effectively reduces cancer cell numbers. Repeated alternating therapy also prevents the outgrowth of resistant clones, in contrast to repeated sequential therapy. The number of cycles of each therapy is indicated.

As expected, treatment with an epithelial-specific chemotherapy enriched for the more-mesenchymal clones, whereas treatment with a mesenchymal-specific chemotherapy enriched for the more-epithelial clones; both treatments selected for clones with reduced phenotypic plasticity. In contrast, a combination therapy that sequentially applied the epithelial- and mesenchymal-specific treatments enriched for clones with increased phenotypic plasticity, with a maximal enrichment for clones that were equal mixtures of cells in the epithelial and mesenchymal states (figure 5*a*). In addition to selecting for clones with increased plasticity, this combination therapy was also significantly more effective at reducing tumour burden relative to either monotherapy (11- to 23-fold; figure 5*a*).

Some of these effects could be magnified by increasing the number of cycles of combination chemotherapy. As the number of chemotherapy cycles was increased from one to three, there was an increase in the enrichment of clones with higher plasticity (figure 5*b*). Additionally, we observed increased tumour sizes with longer simulations, as treatments typically failed to prevent the outgrowth of few faster-growing clones. This was reflected in a dramatically increased variation in the number of surviving cancer cells across simulations.

We found that it was possible to enrich for any given phenotypic plasticity by altering the design of the combination chemotherapy. For example, if seven treatments with an epithelial-specific agent were combined with one treatment with a mesenchymal-specific agent (instead of the three : three design considered above), there was a further enrichment of more-mesenchymal clones (figure 5*c*). Conversely, if seven treatments with a mesenchymal-specific agent were combined with one treatment with an epithelial-specific agent, the most strongly enriched clones were more-epithelial. Increasing the treatment imbalance only magnified this effect. However, the most effective combination therapies were balanced in treatment, and selected for clones with roughly equal mixtures of epithelial and mesenchymal cell types (figure 5*c*). These observations indicated that plasticity was a clonal phenotype that could be selected for (or against) by sequentially applying selection pressures for specific phenotypic states.

We next simulated how combination therapies that sequentially applied epithelial- and mesenchymal-specific treatments compared with therapies that alternated these treatments, while leaving unchanged the total dose of each therapy applied. Although the total dose of therapy applied stayed the same, a combination therapy that alternated between the mesenchymal- and epithelial-specific treatments was far more effective (48-fold) at reducing tumour size relative to the sequentially applied combination, while simultaneously greatly reducing the selection for clones with increased phenotypic plasticity (figure 5*d*). Moreover, we found that doubling the rate at which the therapies were alternated—while halving their durations so as to maintain the same total dose of therapy applied—further reduced tumour size (figure 5*d*). In contrast to the repeated sequential therapy design, repeating the alternating designs did not result in an increased tumour size, suggesting that it prevented the enrichment of resistant clones. This observation demonstrates that the design of combination therapies has an enormous influence on their effectiveness, even in contexts where the total dose of therapy applied remains the same.

## 3. Discussion

In this study we used DNA barcodes to assess phenotypic plasticity across hundreds of clones in a single population of cancer cells. We found that the majority of cancer cell clones give rise to progeny in both the epithelial and mesenchymal states, and the ratio of epithelial and mesenchymal progeny differs between clones. Our results show that this ratio is stable within a clone, even over the course of weeks and through the rigors of single-cell cloning.

We speculate that the marked stability of phenotypic ratios across many generations could be determined by genetic factors, as has been previously proposed [36]. Differences in many such factors across clones would explain the log-normal distribution of phenotypic ratios we observed, if each factor had a small multiplicative effect on phenotypic ratio. As we found that each phenotypic state is a mixture of mostly the same clones, despite the bias of clones towards one state or another, it is not surprising that others rarely saw genetic differences between populations sorted by phenotype [24–27].

Phenotypic switching can serve as a bet-hedging strategy allowing the survival of clones in diverse environments [37]. Phenotypic switching is prevalent across a variety of organisms, including prokaryotes [38,39], yeasts [40,41] and cancer cells [42]. In these examples, phenotype switching allows a clone to sample multiple phenotypes with different sensitivities and resistances, allowing the clone to survive in changing conditions. Since the epithelial and mesenchymal phenotypes we studied here are known to correlate strongly with sensitivity to most cancer therapies [43–45], phenotypic switching between these states would serve as an effective bet-hedging strategy for cancer cells. To be sure, cancer cells are not switching phenotypic states out of an awareness that this strategy will prove beneficial to them. Rather, as indicated by our simulations, cancer cell clones that undergo phenotypic switching have a competitive advantage and thus undergo a selective expansion when treated sequentially with therapies that selectively target the mesenchymal and epithelial states. The diversity of phenotypic plasticities observed across clones allows fluctuating environments to select for a subset of clones with bet-hedging strategies optimally suited to a particular environment. Thus, stably inherited differences in phenotypic plasticity enable tumours to evolve optimal bet-hedging strategies. Phenotypic switching is a powerful mechanism for overcoming selection pressures that vary over time—e.g. chemotherapy regimens—and is consistent with observations of changing phenotypic proportions in progressing tumours [11].

Supporting this interpretation, the enrichment of a particular set of clones based on cell state due to drug-induced selection has been observed *in vitro* [28]. Resistant clones of the HCC827 non-small cell lung cancer cell line were observed to display a more-mesenchymal phenotype than the parental cell line, suggesting that a heritable difference in cell state resulted in their expansion during selection.

In principle, the differences in cell-state proportions we observed in a patient's glioblastoma could arise in the absence of cellular plasticity if the clones identified by our analyses consisted of sub-clones with stable and distinct phenotypes. However, we consider this unlikely since the existence of cellular plasticity in glioblastomas has been supported by several single-cell RNA sequencing studies of patient tumours [18]. While we used gene copy number differences to distinguish between the various clones, our analyses did not assess if these copy number distinctions played a functional role in determining clonal phenotypes.

The stable phenotypic plasticity of clones has implications for the design of combination treatments with phenotype-selective compounds. As conventional chemotherapeutics can cause the enrichment of a mesenchymal, resistant population [10,43,46], there have been significant efforts to develop therapies that target the resistant mesenchymal cells [47,48]. Once developed, implementation of an appropriate treatment regimen will be important for the therapeutic success of these compounds. Even comparing combination therapies with the same total doses, our simulations showed the order and schedule of doses have profound effects on the effectiveness of the therapy. Strikingly, the most simple combination therapy schedule (one treatment, followed by the other) was also the worst performing, while more-rapid, repeated alternations between treatments were far more effective at reducing tumour burden. While changing selections enriched for more plastic clones, we found that even more-rapid alternation would reduce clonal enrichment. These simulations suggest that, without due consideration of treatment schedule, the effectiveness of novel combination therapies could be undervalued. Additionally, our simulations underscore the importance of understanding heterogeneity and recommend alternations to be the most effective combination therapy.

## 4. Material and methods

### 4.1. Barcode library construction

Barcodes were synthesized as oligonucleotides from IDT (Coralville, IA), and are listed in the electronic supplementary material, table S3 as ClonalBarcode5 and ClonalBarcode3. The oligonucleotides were annealed and ligated into pBabe Puro (Addgene #1764, Addgene, Cambridge, MA) that had been digested with BamHI-HF (New England Biolabs) and EcoRI-HF (New England Biolabs), treated with calf intestinal phosphatase (NEB), and purified with a PCR purification kit (Qiagen). One microlitre of 150 nM annealed clonal barcode was ligated to 190 ng of digested pBabe Puro using T4 ligase (New England Biolabs) overnight at 16°. The ligation product was purified using 1× volume of AMPureXP beads (Beckman Coulter) as per the manufacturer's protocol, and eluted into 20 µl. Four times, 2 µl of purified ligation product was transformed into 40 µl of DH5α Electromax *Escherichia coli* (Fisher Scientific). Transformed bacteria were allowed to recover in 1 ml SOC medium, pooled and plated on LB Agar with 100 µg ml^−1^ Ampicillin in two 245 mm plates (Corning, Corning, NY). Some transformed mixture was diluted and plated for counting and colony estimation; this yielded an estimate of approximately 1.7 × 10^6^ colonies. After overnight growth at 37°C, colonies were scraped off and plasmid DNA was extracted with a Gigaprep kit (Qiagen).

### 4.2. Cell culture, virus preparation and infection

MDA-MB-157 cells (ATCC, Manassas, VA) and HEK293T cells were cultured in DMEM supplemented with 10% fetal bovine serum, Penicillin and Streptomycin and GlutaMax (Thermo Fisher Scientific). Viral barcoding vectors were transfected into subconfluent HEK293T cells with pCL-10A1 retroviral packaging plasmid using Fugene 6 (Promega, Madison, WI) and viral supernatant was collected and concentrated with polyethylene glycol (PEG). For concentration, viral supernatant was spun at 931 gravities for 4 min and decanted into 1/5.5 volumes of sterile 50% PEG-3350 in phosphate-buffered saline (PBS). After an overnight 4° incubation, the mixture was spun for 1455 gravities for 20 min, decanted, spun again at 524 gravities for 4 min, and the pellet resuspended in PBS with 1% bovine serum albumin and frozen at −80°C. For infection, 1 × 10^4^ cells were incubated with concentrated virus and 30 µg ml^−1^ protamine sulphate and spun at 1455 gravities for 1.5 h. Viral concentration was optimized to infect approximately 10% of cells. After 48 h, cells were selected with 3 µg ml^−1^ puromycin to kill uninfected cells. Barcoded cells were expanded without discarding cells until the population was at least 2 × 10^7^ cells, and subsequently split into subpopulations no smaller than 2 × 10^6^ cells to maintain clonal representation.

### 4.3. Amplification and sequencing of barcode plasmid pool

Barcodes were amplified using PCR from 2 ng of plasmid with 20 cycles of amplification, using ClBc_5_primer_AAG and ClBc_3_primer_CCT (see electronic supplementary material, table S3). PCR products were run on a 2% agarose gel, extracted using a gel extraction kit (Qiagen), and sequenced on a HiSeq 2000 (Illumina), TruSeq-DNA adaptors. The sequencing primer used was ClBc_seq_primer (see electronic supplementary material, table S3). Sequence data were analysed with a custom Python script that first filtered by quality, where reads were only accepted if they contained fewer than 14 base pairs with a quality score <25, and had no base pairs with a quality score <10. Additionally, reads were only accepted if they contained the index sequences marking each library and the sequences common to every barcode, and every base in those sequences had a quality score of >25. This resulted in 2.4 × 10^6^ reads.

### 4.4. Estimation of plasmid pool complexity

Pool complexity was estimated based on published methods of estimating the number of classes based on sample coverage [49]. Where *N* equals the estimated number of barcodes, *n* = the sample size (2 447 204 reads), *D* = the number of unique barcodes observed (1 530 822), and *f*_1_ = the number of barcodes observed only once (989 844), the sum of the probabilities of observed classes of barcodes was estimated as 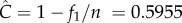. This was used to estimate a lower bound on the number of unique barcodes in the plasmid pool, as 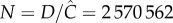.

To estimate the accuracy of this estimation, random sequences of complexity *N* were randomly sampled (with replacement) *n* times, and the count of each unique sequence in the sample determined.

### 4.5. Poisson modelling of viral infection

Viral infection of cells was modelled based on the multiplicity of infection and assuming that the number of infections per cell followed a Poisson distribution, as has been observed by others [50,51]. Multiplicity of infection (MOI) was estimated from the estimated number of cells infected (1.3 × 10^3^ out of 1 × 10^4^); where *m* = MOI and *P*(*n*) = the proportion of cells infected with *n* viruses, the MOI was estimated from 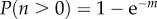 [50]. The MOI was therefore estimated as 0.139. A Poisson PDF was calculated from using this MOI as the *μ* parameter, and was used to estimate the number of cells infected with different numbers of barcodes.

### 4.6. Probability calculations of all cells uniquely barcoded, and simulations of barcodes in multiple cells

The probability at least two cells share a barcode after infection, or *P*(*A*), was calculated as 1 − *P*(*A*′), where *P*(*A*′) is the probability that all cells have unique barcodes. This calculation is analogous to the so-called ‘Birthday Problem’ [52]. Where *N* = the estimated number of barcodes (from sequencing the barcode plasmid pool, 2 570 562) and *c* = the number of cells infected (1372),
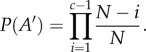


To estimate the probability of different numbers of cells sharing barcodes with other cells, *c* barcodes were randomly sampled from *N* barcodes, 5 × 10^5^ times with replacement.

### 4.7. Intracellular flow cytometry

Cells were trypsinized, washed in DMEM supplemented with 10% fetal bovine serum (Sigma-Aldrich, St. Louis, MO), washed 2× in PBS, and spun (as with all subsequent washes) at 524 gravities for 3 min. The pellet was disrupted by vortexing and the cells fixed by dripping in 2 ml of ice-cold 70% ethanol while vortexing. Vortexing was continued for 30 s and the cells incubated overnight at 4°C. Cells were blocked by washing 3× in FACS buffer (FB), consisting of PBS supplemented with 6% fetal bovine serum (Sigma-Aldrich). Cells were filtered through a 40 µm filter, counted on a haemocytometer, resuspended to 1 × 10^6^ cells ml^−1^ in FB and stained with a 1 : 50 dilution of mouse anti K8/18, clone C51 (Cell Signaling, Danvers, MA), for 1–2 h on ice. After washing 3× with FB, cells were stained in FB at a concentration of 1 × 10^6^ cells ml^−1^ and a 1 : 1000 dilution of goat anti mouse Fab Alexa Fluor 488 (Cell Signaling), incubating for 0.5 to 1 h on ice in the dark. Cells were washed 3× in FB and resuspended at 1 × 10^6^ cells ml^−1^ in FB. Samples were run on a Fortessa (Becton Dickinson, Franklin Lakes, NJ), and flow cytometry data were analysed with FlowJo (Tree Star, Ashland, OR).

### 4.8. Fluorescence-activated cell sorting

For single-cell cloning, clonally barcoded MDA-MB-157 cells were trypsinized, washed with PBS supplemented with 3% FBS, sent through a 40 µm filter, and resuspended to 1 × 10^6^ cells ml^−1^. A FACSaria (Becton Dickinson) was used to sort single cells into wells of 96-well plates, each well containing 100 µl of DMEM with 10% FBS.

After expansion of the barcoded population of cells, a portion of the cells were stained for keratin 8/18 expression and sorted via FACS. Portions were separated out of the population and sorted at three time points each separated by a week (day 0, day 7, day 14).

For these sorts based on keratin 8/18 expression, cells were stained as in intracellular flow cytometry, but stained at 1 × 10^7^ cells ml^−1^ in FB and with a 1 : 60 dilution of mouse anti K8/18, clone C51 (Cell Signaling), for 1–2 h on ice. After secondary staining and washes, cells were resuspended at 1 × 10^7^ cells ml^−1^ in FB and sorted on a FACSAria (Becton Dickinson) set to maximize yield. Sorted samples were analysed on the FACSAria to measure the proportion of cells mis-segregated, counted on a haemocytometer, and split in half. Barcodes were extracted from these cells as described below.

### 4.9. Extraction and amplification of barcodes from genomic DNA

Genomic DNA was collected with a DNeasy kit (Qiagen, Venlo, The Netherlands). All genomic DNA was digested with BamHI and EcoRI (New England Biolabs, Ipswich, MA), using 3 units µg^−1^ and digesting for 1 h at 37°. Digested DNA was directly purified from solution with a gel extraction kit (Qiagen), and barcodes size-selected with Agencourt AMPure XP beads (Beckman Coulter, Brea, CA). For size selection, one-half volume of beads was added to the DNA mixture to bind to large DNA fragments, mixed by vortexing, and incubated at room temperature for 5 min. After precipitating the beads with a magnet, the supernatant containing small DNA was removed, and DNA was purified from the supernatant with a gel extraction kit (Qiagen) and quantified on a Nanodrop (Thermo Scientific). Barcodes were amplified from purified size-selected DNA using 25 cycles of PCR with ExTaq (Takara Bio, Kyoto, Japan), assembling the reaction mixture on ice. Template was added to a final concentration of 10 ng µl^−1^, and all size-selected DNA was used as template. This PCR step was used to also add library-specific index sequences (to allow for sequencing multiple samples in the same sequencing lane) and adaptor sequences for high-throughput sequencing. Index sequences were designed to have at least two differences from all other index sequences. Primer sequences are listed in the electronic supplementary material, table S3. PCR products were purified with a PCR purification kit (Qiagen). Samples of 4, 2 and 1 µl of each PCR product were run on a 2% agarose gel and the intensity of the 131 base pair band quantified electronically. Samples' relative DNA concentration was computed with linear regression and the samples were combined in equimolar ratios. This combined library was run on a 2% agarose gel, and the 131 base pair band was purified with a gel extraction kit (Qiagen). The purified band was sequenced on a HiSeq2000 (Illumina, San Diego, CA); the resulting sequencing data are available at the NCBI SRA (SRX1175944).

### 4.10. Analysis of sequencing data (sorted cells)

Sequencing data were analysed with custom Python scripts. Reads were first filtered by quality, where reads were only accepted if they contained fewer than 6 base pairs with a quality score < 25, and had no base pairs with a quality score < 15. Additionally, reads were only accepted if they contained the index sequences marking each library and the sequences common to every barcode. These steps reduced 1.67 × 10^8^ reads to 1.01 × 10^8^ reads. This quality filtering procedure was more stringent than that used to analyse barcodes from the plasmid pool due to the increased cycles of amplification involved in library construction and lower starting pool complexity, which resulted in lower quality reads. Reads were then separated based on library-specific sequences that were introduced during PCR to distinguish samples. Taking reads for barcodes seen at least twice, we, as others, combined reads that could be connected with few mismatches, using the most abundant barcode to represent the group and giving it the abundance of the sum of the group's reads [29,53]. To avoid erroneously combining barcodes that were by chance similar in sequence, we repeatedly iterated down the list of barcodes ordered by abundance, grouping together less abundant barcodes that were within one mismatch, and then repeating the process grouping together less abundant barcodes within two, three and four mismatches. We then removed from analysis any barcodes that were not detected in any libraries from one or more time points.

These data were used to test for clones' bi-lineage potential and cell-state bias, below, to allow for statistical analysis of clones based on the actual number of reads.

For further analysis, reads for each library were normalized by dividing by the sum of reads for that library multiplied by the fraction of the population consisting of that cell state at the time of sorting, being 60% for K8/18 high and 40% for K8/18 low. Any barcodes not found in a library were given a fractional value of 1 × 10^−6^ for that library. To deal with sort contamination, for each barcode, and for each time point, we subtracted from each sorted library the average fraction of total reads of the other sorted populations multiplied by the fractional contamination observed in that sort from post-sort flow cytometry. Any barcode abundance thus brought to less than zero was given a value of 1 × 10^−6^. After determining in this way the size of each clone in each state, the results from the two sequenced replicates from each time point (see above) were combined by taking their mean.

### 4.11. Testing for clones' bi-lineage potential

To determine whether clones did in fact have bi-lineage potential, we asked whether, for each clone, we could reject the hypothesis that there were as many reads as could be expected via sort contamination (mis-sorted cells), assuming each clone was composed entirely of cells in one state. The proportion of mis-sorted cells was determined via flow cytometry of the sorted cells (see above), here represented as *σ_m_*_,*t*_ for the proportion of mesenchymal-sorted cells at time *t* that were actually mis-sorted epithelial cells, and similarly *σ_e_*_,*t*_.

After sorting at three time points (days 0, 7 and 14; see above), each pool of sorted cells was split into two, and sequenced (see above). At each time point, the reads for each clone in each state were summed, creating *r*_e_(*c, t*) for the summed epithelial (keratin 8/18+) reads of clone *c* at time point *t,* and similarly *r*_m_(*c, t*). As the count of observed reads for each clone was being compared with reads expected from sort contamination, the un-normalized reads from clones were used (see above).

In order to test for bi-lineage potential, we calculated for each state, at each time point, the expected probability of a read in the other state from sort contamination, assuming all cells were in the first state, or *pC*_e_ (*t*) for the probability of epithelial reads being mis-called as mesenchymal, assuming all cells were epithelial, and *pC*_m_ (*t*) for the probability of mesenchymal reads being mis-called as epithelial, assuming all cells were mesenchymal.

For ease of understanding the calculation of these probabilities, the reads from the epithelial-sorted population can be visualized as a combination of reads from real epithelial cells (totalling (1 − *σ_e_*) times the sum of epithelial-sorted reads) and reads from real mesenchymal cells (totalling *σ_e_* times the sum of epithelial-sorted reads). Similarly, the reads from the mesenchymal-sorted population can be viewed as a combination of reads from real mesenchymal cells (totalling (1 − *σ_m_*) times the sum of mesenchymal-sorted reads) and reads from real epithelial cells (totalling *σ_m_* times the sum of mesenchymal-sorted reads).

Therefore, the sum of reads from correctly sorted epithelial cells (*E*) at time *t* is
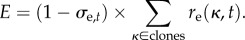


The sum of reads from incorrectly sorted epithelial cells (*E*′) at time *t* therefore is
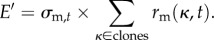


The sum of reads from correctly- and incorrectly-sorted mesenchymal cells was calculated similarly.

*pC*_e_ is defined as the proportion of all epithelial reads that were mis-called as mesenchymal, which is equal to *E*′/(*E* *+* *E*′). Therefore, these probabilities were calculated as
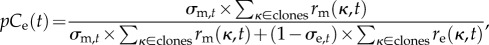
and
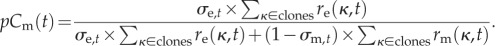


For each time point, and each clone, these probabilities were used to test the hypothesis that each clone was actually monolineage. To test the null hypothesis that all clones were epithelial, each clone was evaluated at 1 − the CDF of a binomial distribution with *p* = *pC*_e_ and 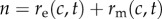 for clone *c,* evaluated at *x* = *r*_m_(*c*,*t*). Similarly, to test the null hypothesis that all clones were mesenchymal, each clone was evaluated at 1 − the CDF of a binomial distribution with *p* = *pC*_m_ and 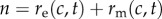 for clone *c,* evaluated at *x* = *r*_e_(*c*,*t*). These resulting *p* values from all states and all time points were corrected for multiple hypothesis testing using the Benjamini–Hochberg method [54]. The null hypothesis that a clone was monolineage at a particular time point was rejected to control the false discovery rate (FDR) at 0.05. A clone was declared monolineage if the null hypothesis was rejected at all time points for the same state, and at no time points for the other state. A clone was declared bi-lineage if the null hypothesis was rejected in both states (still at the FDR of 0.05) in at least one time point.

### 4.12. Testing for clones' cell-state bias

To test whether clones had bias in cell state, we attempted to reject the null hypothesis that each clone's cell-state proportions matched the population cell-state proportion. Again, pre-normalized reads (see above) were used. Reads from the two sequenced replicates of each sorted population were summed. For each clone, at each time point, the expected number of reads in the epithelial and mesenchymal states were calculated, or *E*_e_(*c*,*t*) and *E*_m_(*c*,*t*), respectively:
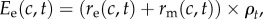

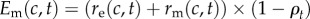

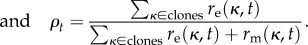


The *χ*^2^ test was used to examine the significance of the fit between the observed and expected reads. The upper CDF of the *χ*^2^ distribution with one degree of freedom was evaluated at *x,* where:
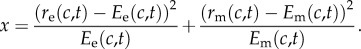


These resulting *p* values from all time points were corrected for multiple hypothesis testing using the Benjamini–Hochberg method [54]. The null hypothesis that a clone at a time point had a cell-state bias matching the population cell-state proportions was rejected so as to control the false discovery rate at 0.05. Clones were declared significantly different from the population cell-state proportion if this null hypothesis was rejected at all time points.

### 4.13. Fraction epithelial and log_2_ (epithelial/mesenchymal) ratio calculations

After normalizing the sequencing data of sorted cells (see above) to determine the size of each clone in the epithelial and mesenchymal states, and after subtracting those reads estimated to come from sort contamination, each clone's cell-state bias was determined, represented by the fraction of the clone that was epithelial (fraction epithelial) or the log_2_(epithelial/mesenchymal) ratio (log_2_ (*E*/*M*)).

Here, *E_c,t_* equals the normalized fraction of cells of clone *c* in the epithelial state at time point *t*, and similarly *M_c,t_*. These estimated cell counts are normalized such that the sum of epithelial and mesenchymal counts across clones at each time point equals one. For both of these calculations, dividing *E* by *M* or (*E* + *M*) cancels out this normalization factor, rendering the calculations equivalent to those using counts of cells. The fraction of clone *c* epithelial at time point *t* was calculated as
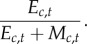
The log_2_ (*E*/*M*) ratio for clone *c* at time point *t* was calculated as
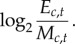
The log_2_(*E*/*M*) ratio for clone c averaging across the three time points was calculated as
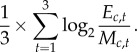


### 4.14. Testing the significance of the correlation of clones' cell-state bias across time points

The Pearson correlation of clones' log_2_(*E*/*M*) ratio across time points was determined to assess the stability of cell-state bias. To determine the probability of randomly obtaining a correlation higher than the one observed in each of the three comparisons across time points, the barcode labels of one time point in each comparison were randomly shuffled. After each randomization, the Pearson correlation was evaluated and the correlation coefficient *ρ* recorded. After 1 × 10^6^ such randomizations, the distribution of randomized *ρ* values was compared with the observed *ρ*, and the proportion of randomized *ρ* greater than observed *ρ* determined.

### 4.15. Clone growth rate calculations

From the frequency of splitting during cell culture, the population of cells was estimated to double approximately three times per week. The population of cells sorted at the first time point consisted of 2.9 × 10^7^ cells, and this population growth rate was used to estimate the number of cells at 1 and 2 weeks later, at the second and third time points. As the barcode sequencing information was used to calculate relative size of each clone as a fraction of the total population, these population cell numbers were used to compute the cell numbers of each clone at each time point through multiplication. Each clone's number of cells at the second and third time points (*N_i,c_*) were compared with cell numbers from the first time point (*N*_0,*c*_) to compute each clone's growth rate over these two intervals; the two rates were averaged to compute each clone's growth rate (*k_c_*):
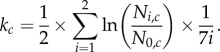


### 4.16. Calculation of Shannon entropy of the distribution of clones' epithelial/mesenchymal ratio

After calculating the geometric-average log_2_ (epithelial/mesenchymal) for each clone across the three examined time points, clones were binned from the minimum ratio to the maximum ratio in bins of width 1 (corresponding to a two-fold change in ratio). The Shannon entropy of clones thus binned was calculated.

### 4.17. Flow cytometry of single-cell clones

Single-cell clones were trypsinized, washed in DMEM supplemented with 10% fetal bovine serum (Sigma-Aldrich), and washed 2× in PBS. Cells were fixed as in intracellular flow cytometry. To serve as an internal staining control for the single-cell clones, pooled clones (the parental barcoded population) were fixed in the same way as the single-cell clones. This pooled clone population was resuspended at 1 × 10^6^ cells ml^−1^ in PBS and covalently stained with 1 µl ml^−1^ of Blue Live/Dead Discrimination Dye (Thermo Fisher Scientific, Cambridge, MA) for 30 min on ice in the dark. Cells were blocked by washing 3× in FACS buffer, filtered through a 40 µm filter, counted on a haemocytometer, and resuspended to 1 × 10^6^ cells ml^−1^ in FB. For analysis of single-cell clones, clones were mixed 1 : 1 with samples from the covalently stained pooled clones. Samples were then stained as in the intracellular flow cytometry protocol and run on a Fortessa (Becton Dickinson), and the flow cytometry data analysed with FlowJo. After using FlowJo gating to remove debris, the flow cytometry data were exported and analysed with a custom Python script. In brief, this script separated the cells of the pooled clones, and determined thresholds of K8/18 staining to gate E and M from this population such that the gates contained the same proportion of cells as the gates used for cell sorting. These gates were then used to determine the proportion of cells from the single-cell clone that would have been sorted as epithelial or mesenchymal to calculate the log_2_ (epithelial/mesenchymal).

### 4.18. PCR and Sanger sequencing of barcodes from single-cell clones

Genomic DNA was collected with a DNeasy blood and tissue kit (Qiagen) as per the manufacturer's instructions. Barcodes were amplified with nested PCR, using two sets of primers (IDT, Coralville, IA) (first ClBc_A5 and ClBc_A3, then ClBc_B5 and ClBc_B3; see electronic supplementary material, table S3) to specifically amplify one band. The initial genomic DNA concentration was 1.2 ng µl^−1^, and each round of PCR was 25 cycles. The PCR product was purified with a PCR purification kit (Qiagen) and sequenced with Sanger sequencing (Genewiz, South Plainfield, NJ).

### 4.19. Analysis of correlation of single-cell clone/pool phenotypic ratio

Narrow-sense heritability was calculated as the Pearson correlation coefficient [55]. Twenty-eight clones were deemed sufficient as power analysis showed a power of 0.96 to reject the null hypothesis at a significance of 0.01 for correlations of 0.7, calculated using the pwr package in R. To determine the probability of randomly obtaining a correlation higher than the one observed between single-cell clones' phenotypic proportion and those clones' phenotypic proportion in the pooled experiment, the barcode labels of single-cell clones were randomly shuffled between the single-cell clones using a custom script. After each randomization the Pearson correlation was evaluated and the correlation coefficient *ρ* recorded. After 1 × 10^6^ such randomizations, we compared the distribution of randomized *ρ* values with the observed *ρ*.

### 4.20. Glioblastoma single-cell RNA sequencing data

Normalized single-cell RNA sequencing data from primary glioblastoma tumours were obtained from the Gene Expression Omnibus (accession GSE57872) [18].

### 4.21. Copy-number estimation from single-cell RNA sequencing data and clone separation

As has been previously described [18,34], changes in copy number were estimated through analysis of single-cell RNA expression by chromosomal location. Mean normalized (by gene across cells) log_2_(TPM + 1) RNA values from single cells were accessed from GSE57872 [18,56]. RNA data were from cells that were identified either as non-cancer cells or as cells from tumour MGH31. For the purposes of determining copy-number variation, these data were thresholded, so that values greater than were set to 3, and values less than −3 were set to −3. For each cell, we computed a sliding average of the expression of 101 genes moving down the list of genes with RNA data ordered by chromosomal location, to build a copy-number variation profile (CNV profile). We then centred each cell's CNV profile at 0 (subtracting from each profile the mean value) to deal with any differences in expression remaining across cells. This meant that the CNV profile value for cell *j* at position *i* (CNV*_i,j_*) is
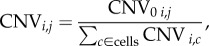
where
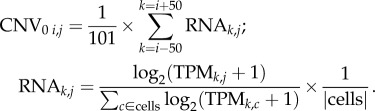


To normalize to the average expression by chromosomal location, so as to deal with differences in expression across chromosomes, each cell's CNV profile was normalized using an averaged CNV profile from normal cells (CNV_Base_), computed for each genomic location through an identical sliding-average strategy from normal neural cells identified in the same RNA-seq dataset [18]. For each cell, this normalized CNV (CNV_norm_) was calculated as follows:
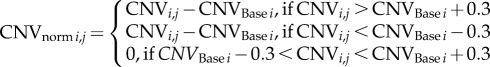


In this way, CNV values were only recorded if they deviated significantly from the value obtained from normal cells, where a difference of 0.3 corresponds to a 23% change.

The cells' normalized CNV profiles were clustered via Ward's method, using the Euclidean distances between CNV profiles. This method clusters vectors by finding, at each step, the pair of clusters that leads to the minimum increase in within-cluster variability when the clusters are combined. In this way, the hierarchical clustering of CNV profiles clustered cells based on similar CNV profiles; cells were divided into clones based on this hierarchical clustering.

### 4.22. Subtype and stemness classification from single-cell RNA sequencing data

Cells were classified by subtype as previously described [18]. Cells were scored by subtype using published lists of genes enriched in each subtype [35], or marking cells with increased stemness [18]. Mean normalized (by gene, across cells) log_2_ (TPM + 1) RNA values for single cells were accessed from GSE57872 [18,56]. For each cell, a score was calculated for each subtype. These scores for cell *i* for subtype *j* (*S_i,j_*) were calculated by taking the average expression of classifier genes for subtype *j* (*G_c_*) in cell *i,* and subtracting the mean expression of every gene in cell *i*:
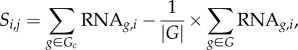

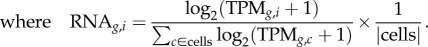


To evaluate each individual score for significance, we adapted a previously published method [18], evaluating the enrichment of each score relative to random sets. For each subtype, we made 100 random subtype-classifier gene sets from randomly sampling the set of sequenced genes. Each random set had the same number of genes as the real subtype gene set. We called each cell's subtype score as enriched or depleted by comparing it to these random scores. If the real score was greater than 95% of the random scores, we called it enriched, whereas if the real score was less than 95% of the random scores, we called it depleted.

To determine whether clones had different distributions of subtype scores, suggesting differences in plasticity, we evaluated the distributions of subtype scores across cells grouped into clones by the clustering above with a Kruskal–Wallis test.

### 4.23. Simulations

Mechanistically, a ‘tumour’ was seeded with 500 clones. Each clone was assigned a fraction epithelial, growth rate and cell numbers matching a randomly chosen observed clone, so as to simulate the distribution of the observed growth rates and fraction epithelial. Both the growth rate and fraction epithelial parameters were inherent to the clone for the entirety of the simulation. Each clone's starting cell numbers were drawn from those observed at day 0 of sorting (see above). After instantiation of the tumour, growth was modelled under different treatment regimes, with 15 time points modelling a day. The amount of cells for each clone after division were calculated as: 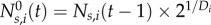, where *N_s,i_*(*t*) represents the number of cells of clone *i* in state *s* at time *t, D_i_* represents the doubling time (in the time scale of the simulation, where 15 time points is equivalent to 1 day) of clone *i*.

At each time point, cells were also allowed to differentiate. Each clone's transition probabilities for going from epithelial to mesenchymal or mesenchymal to epithelial were defined so that 20% of cells changed state per division, and the ratio of transition probabilities matched the clone's defined equilibrium of cell states; in this way, the clones have stable cell-state proportions at equilibrium and slowly return to equilibrium after the cell-state ratios are perturbed through selection. This probability of differentiation was chosen to reflect those observed in other contexts [12]. The resulting number of cells of clone *i* in state *s* (where the other state is *s*'), or 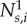, as a consequence of differentiation is as follows:
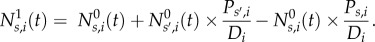


Here, *P_s,i_* represents the probability that a cell of clone *i* differentiates from state *s* to state *s*'. This was calculated as follows, where *R_i_* represents the equilibrium epithelial : mesenchymal ratio of clone *i*: 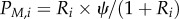 and 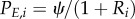; *ψ* here represents the probability of a cell differentiating during a division, or 0.2 as discussed above. If no treatment is simulated during this time point, 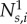 is now the final count of cells for clone *i* in state *s* for time point *t*, or *N_s,i_*(*t*).

Treatments were applied as mesenchymal-specific or epithelial-specific, where a mesenchymal targeting therapy killed a 10-fold higher fraction of the mesenchymal cells compared to epithelial cells. This was chosen to match the relative effectiveness of certain *in vitro* compounds on cells in different differentiation states [57]. The number of cells remaining after death (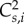) for a treatment targeting state *s* is calculated as 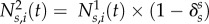, and for a treatment targeting state *s*' (the other state), 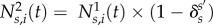, where 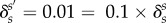. In this case 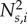 is now the final count of cells for clone *i* in state *s* for time point *t*, or *N_s,i_*(*t*).

Each treatment cycle killed a fraction of the cells for 30 time points, simulating a course of therapy for 2 days, which was followed with 20 time points of no treatment. The simulation was ended after the conclusion of treatment-rest periods; the number and pattern of treatment-rest periods varied among the simulations. A variety of treatment combinations were simulated as detailed in the results. To compare the results of different simulations, clones were binned by their fraction epithelial. Each clone's fold change in cell numbers during the simulation (*fc_i_* for clone *i*) was computed as
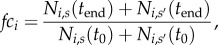
where *N_s,i_*(*t*) represents the number of cells of clone *i* in state *s* at time *t*, *t*_0_ is the time point of the start of simulated treatments, and *t*_end_ the time point of the end of simulated treatments. For each bin of clones by fraction epithelial, the median fold change in cell numbers for the clones in each bin was computed. The sum of cells across clones at the last point was also computed for each simulated treatment. Simulations were repeated 500 times, and the 0.1, 0.5 and 0.9 quantiles of the median clone fold change for each bin across simulations were recorded. Similarly, the 0.1, 0.5 and 0.9 quantiles of the sum of cell numbers across simulations were recorded.

## Supplementary Material

Supplementary Table 1: Phenotypic ratios and estimated population sizes of barcoded clones

## Supplementary Material

Supplementary Figures and Supplementary Tables 2-3

## Supplementary Material

Reads for each clone

## Supplementary Material

Normalized reads for each clone

## Supplementary Material

Empirical data for simulations

## Supplementary Material

Markers for Glioblastoma subtypes and stem-ness

## Data accessibility

Phenotypic ratios and estimated population sizes of barcoded clones can be found in the electronic supplementary material, table S1. The phenotypic ratios of single-cell clones can be found in the electronic supplementary material, table S2. Primers and oligonucleotide sequences used are in the electronic supplementary material, table S3. The number of observed reads for each clone is in electronic supplementary material, clonereads.txt. Normalized reads for each clone (see Material and methods) are in the electronic supplementary material, norm_filtered_read.csv. Analysed data used for clone simulations are available in the electronic supplementary material, observeddata.csv. Markers for determining glioblastoma subtypes [18,35] are in the electronic supplementary material, markerssubtype.txt. Sequencing reads of sorted cell barcodes from this manuscript are in the NCBI short read archive, accession SRX1175944. Single-cell RNA sequencing data were obtained from the NCBI gene expression omnibus at GSE57872 [18]. Codes associated with this project can be found at https://github.com/rmathisWI/CloneCodes.
