# Supplementary material for: Cancer cells exhibit clonal diversity in phenotypic plasticity
Source: Open Biol. 2017 Feb 15;7(2):160283. doi: 10.1098/rsob.160283 (PMC5356442; doi:10.1098/rsob.160283)
Supplement: Supplementary Figures and Supplementary Tables 2-3 [file rsob160283supp2.pdf]

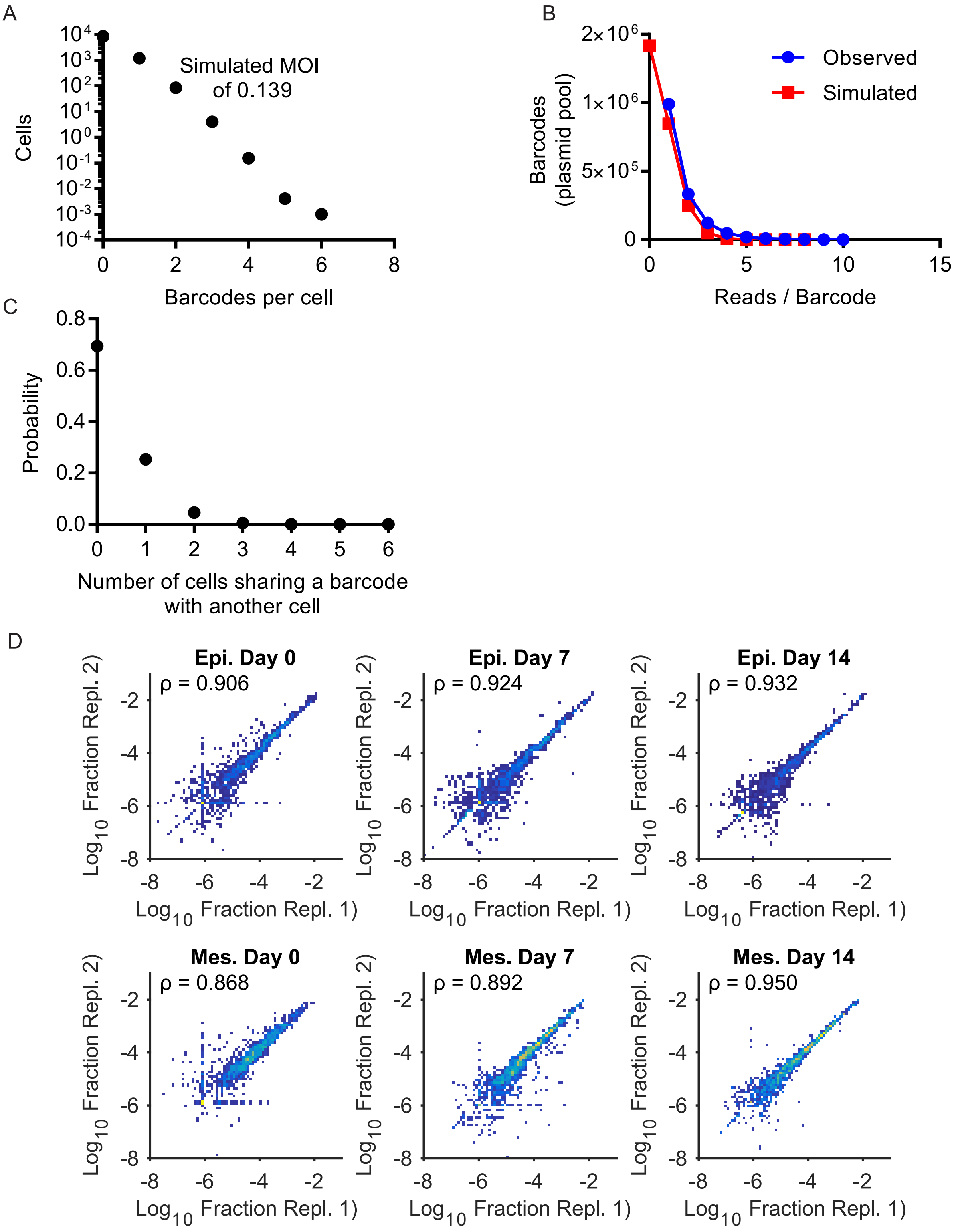

Supplementary Figure 1: Tracking clones with DNA barcodes

(a) Simulation of the infection conditions from the calculated MOI using a Poisson distribution suggests very few cells (6.8%) received more than one barcode. **(b)** High-throughput sequencing results of the pool of barcodes, plotted as the number of barcodes observed with each number of reads vs the reads per barcode. Also plotted is the expected number of unique barcodes sequenced assuming equal abundance of barcodes and $2.6*10^6$ total unique barcodes, the complexity calculated from the observed distribution (see Materials and Methods). **(c)** Plot of the probability (y) of a certain number of infected cells (x) sharing a barcode with another cell (via simulation). **(d)** Technical replicates of barcodes sampled from the same population of labeled cells show good reproducibility. Each barcode's estimated fraction of the total population is plotted in two halves of one population, split before extracting DNA. Barcodes not found in one replicate were given values of $1*10^{-6}$ (see Materials and Methods`). Color indicates the density of points; abbreviations are epithelial cells (Epi.), mesenchymal cells (Mes.), technical replicate (Repl.).

A

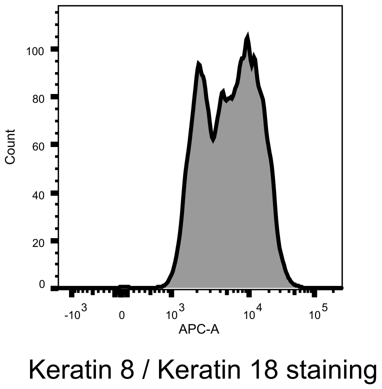

B

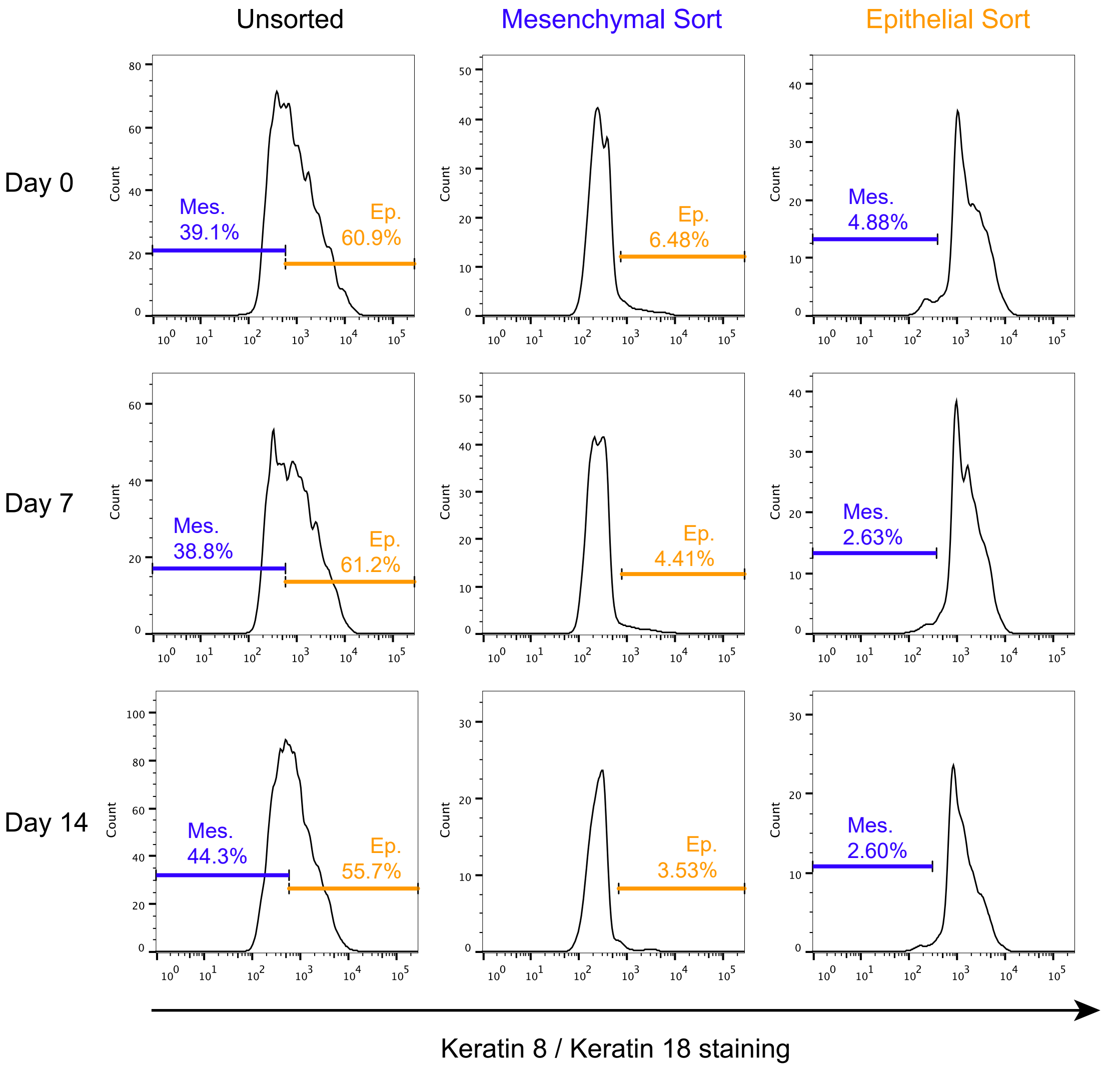

Supplementary Figure 2: Fluorescence activated cell sorting based on keratin expression

(a) Histogram of barcoded MDA-MB-157 cells based on Keratin 8 / Keratin 18 expression as assayed by flow cytometry. **(b)** Post-sort flow cytometry of sorted populations; cells sorted in error were computationally subtracted from the barcode data. Shown are unsorted cells, and each sorted fraction for each time point: Keratin 8 / Keratin 18 high cells (Epithelial, Ep.) and Keratin 8 / Keratin 18 low cells (Mesenchymal, Mes.).

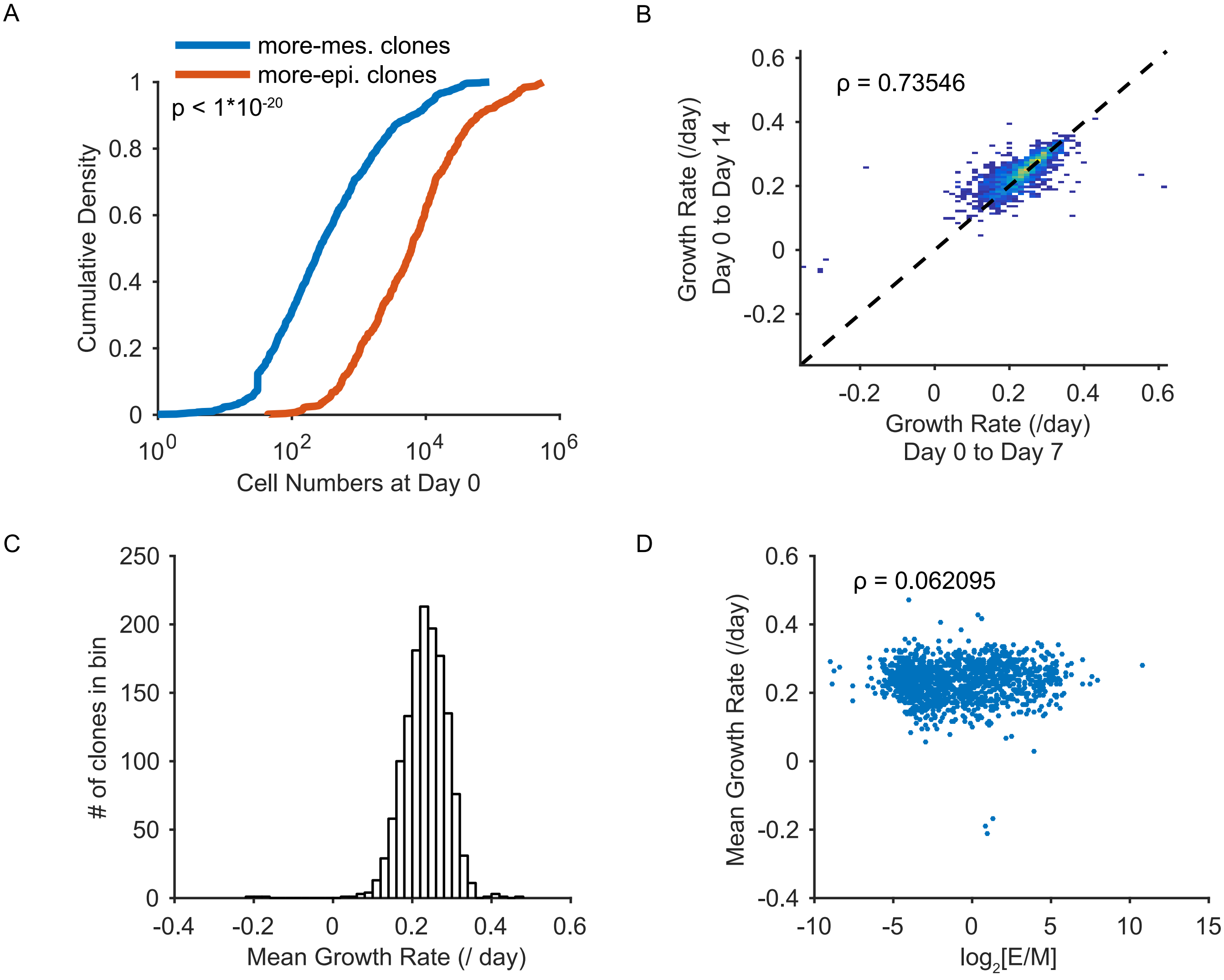

Supplementary Figure 3: Growth rates of clones
(a) Estimated cell numbers at day 0 of more-mesenchymal (more-mes.) and more-epithelial (more-epi.) clones. The p value displayed is from a rank-sum test of these two groups. **(b)** Growth rate (k, per day) of clones, determined from the change in each clone's cell numbers from day 0 to day 7, or to day 14. Each dot corresponds to a clone; overlapping dots produce a different color. The Pearson correlation (ρ) is displayed, and the line y=x is plotted (dotted line). **(c)** Histogram of the average growth rate of each clone. **(d)** Plot of each clone's average growth rate vs its log2(Epithelial/Mesenchymal) ratio (log_2[E/M]). The Pearson correlation (ρ) is displayed.

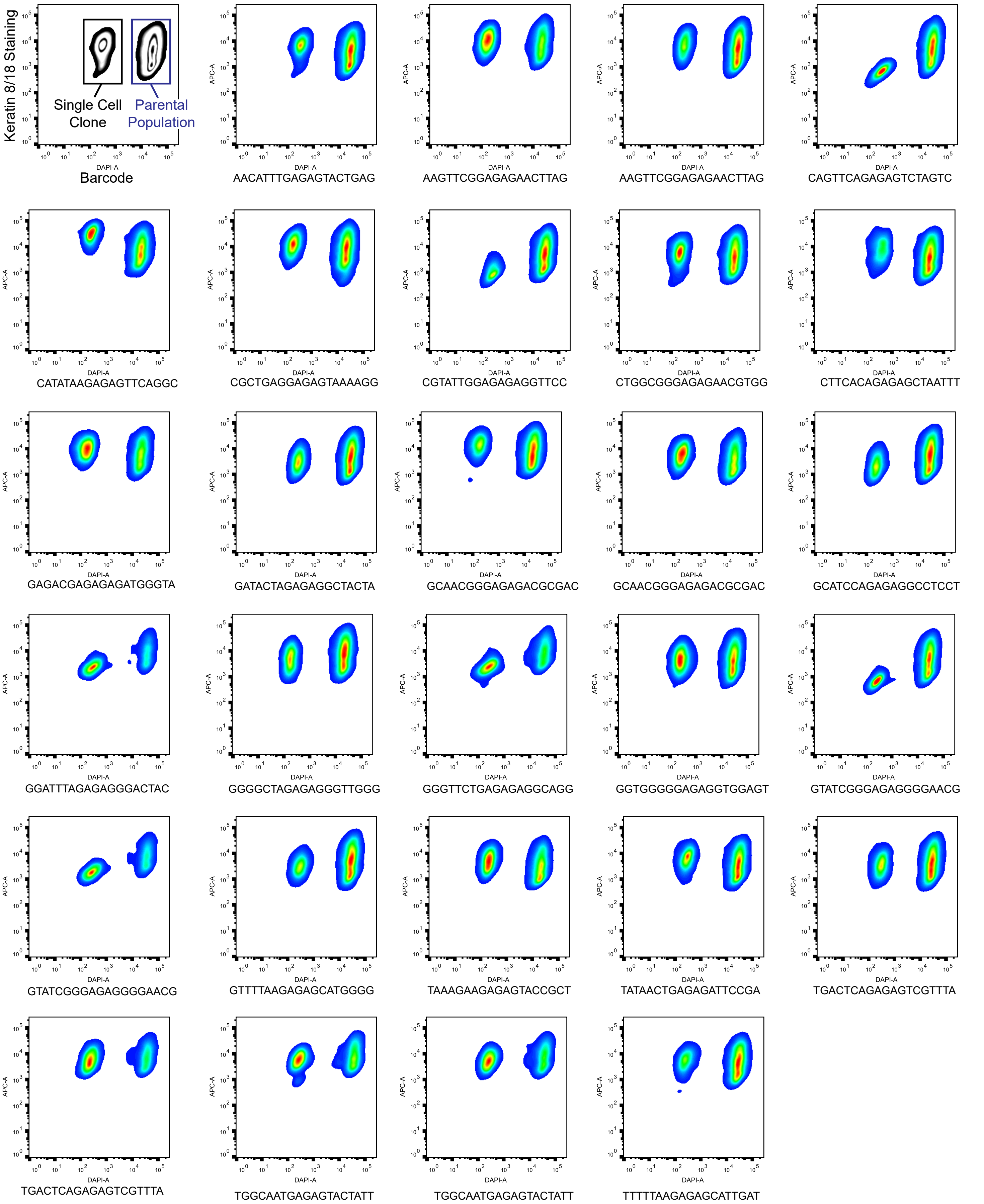

Supplementary Figure 4: Flow cytometry of single cell clones

Flow cytometry of single-cell clones (see Figure 3), with Keratin 8 / Keratin 18 staining on the y axis, and the covalent stain marking the spiked-in polyclonal/parental population on the x axis. The upper-left plot is a key showing the different populations. Each clone's barcode is displayed under each clone.

Barcode	Single Cell Clone $\log_2$[E/M]	Pool $\log_2$[E/M]
AACATTTTACTGAG	3.345	0.891
AAGTTCGAACTTAG	1.687	1.934
AAGTTCGAACTTAG	1.895	1.934
CAGTTCATCTAGTC	-9.522	-3.157
CATATAATTCAGGC	6.583	5.292
CGCTGAGTAAAAGG	2.737	0.817
CGTATTGAGGTTCC	-5.642	-4.257
CTGGCGGAACGTGG	1.932	1.285
CTTCACACTAATTT	2.626	-0.987
GAGACGAATGGGTA	3.329	3.188
GATACTAGCTACTA	-0.825	-1.357
GCAACGGACGCGAC	3.457	1.310
GCAACGGACGCGAC	2.833	1.310
GCATCCAGCCTCCT	-2.721	0.031
GGATTTAGGACTAC	-8.617	-3.929
GGGGCTAGGTTGGG	-1.141	-1.084
GGGTTCTAGGCAGG	-6.932	-4.074
GGTGGGGGTGGAGT	0.504	-0.905
GTATCGGGGGAACG	-9.928	-5.013
GTATCGGGGGAACG	-9.179	-5.013
GTTTTAACATGGGG	-1.584	-2.766
TAAAGAATACCGCT	2.837	1.391
TATAACTATTCCGA	3.105	1.432
TGACTCATCGTTTA	-0.953	1.166
TGACTCATCGTTTA	0.379	1.166
TGGCAATTACTATT	-1.599	1.280
TGGCAATTACTATT	-0.955	1.280
TTTTTAACATTGAT	1.559	1.090

Supplementary Table 2: Phenotypic ratio of single cell clones

Supplementary Table 3: Table of primers and oligonucleotide sequences

ClBc_5_primer_AAG	AATGATACGGCGACCACCGAGTAGACGGAGCGGACAACACTAAGACAGG
ClBc_5_primer_CAT	AATGATACGGCGACCACCGAGTAGACGGAGCGGACAACACTCATACAGG
ClBc_5_primer_TAG	AATGATACGGCGACCACCGAGTAGACGGAGCGGACAACACTTAGACAGG
ClBc_5_primer_CAA	AATGATACGGCGACCACCGAGTAGACGGAGCGGACAACACTCAAACAGG
ClBc_5_primer_TTC	AATGATACGGCGACCACCGAGTAGACGGAGCGGACAACACTTTCACAGG
ClBc_5_primer_ATC	AATGATACGGCGACCACCGAGTAGACGGAGCGGACAACACTATCACAGG
ClBc_5_primer_GTT	AATGATACGGCGACCACCGAGTAGACGGAGCGGACAACACTGTTACAGG
ClBc_5_primer_GTA	AATGATACGGCGACCACCGAGTAGACGGAGCGGACAACACTGTAACAGG
ClBc_3_primer_TCC	CAAGCAGAAGACGGCATACGAGCTCTTCCGATCTGGCTCTGGATTGTCAGCGTTCCCGTGC
ClBc_3_primer_TCG	CAAGCAGAAGACGGCATACGAGCTCTTCCGATCTGGCTCTGGATTGTCAGCGTTCGCGTGC
ClBc_3_primer_GCT	CAAGCAGAAGACGGCATACGAGCTCTTCCGATCTGGCTCTGGATTGTCAGCGTGCTCGTGC
ClBc_3_primer_CCT	CAAGCAGAAGACGGCATACGAGCTCTTCCGATCTGGCTCTGGATTGTCAGCGTCCTCGTGC
ClBc_3_primer_GGA	CAAGCAGAAGACGGCATACGAGCTCTTCCGATCTGGCTCTGGATTGTCAGCGTGGACGTGC
ClBc_3_primer_CGA	CAAGCAGAAGACGGCATACGAGCTCTTCCGATCTGGCTCTGGATTGTCAGCGTCGACGTGC
ClBc_3_primer_AGC	CAAGCAGAAGACGGCATACGAGCTCTTCCGATCTGGCTCTGGATTGTCAGCGTAGCCGTGC
ClBc_3_primer_AGG	CAAGCAGAAGACGGCATACGAGCTCTTCCGATCTGGCTCTGGATTGTCAGCGTAGGCGTGC
ClBc_seq_primer	CCGAGTAGACGGAGCGGACAACACT
ClonalBarcode5	5'phos/GATCCTAGACGGAGCGGACAACACTGACACAGGNNNNNNNGAGAGNNNNNNNGCACGTGTACGCTGACAATCCAGAGCCG
ClonalBarcode3	5'phos/AATTCGGCTCTGGATTGTCAGCGTACACGTGCNNNNNNNCTCTCNNNNNNNCCTGTGTCAGTGTTGTCCGCTCCGTCTAG
CLBc_A5	CTCTGCAGAATGGCCAACC
CLBc_A3	CTTCTGGAATAGCTCAGAGGCCGAG
CLBc_B5	GCACCTTTAACCGAGACCTCATCAC
CLBc_B3	GACTTTCCACACCTGGTTGC
